# The complete mitochondrial genome of Eastern Yellow Wagtail (*Motacilla tschutschensis)*

**DOI:** 10.1080/23802359.2019.1674737

**Published:** 2019-10-11

**Authors:** Xiaodong Gao, Dajie Xu, Tian Xia, Huashan Dou, Weilai Sha, Honghai Zhang

**Affiliations:** a College of Life Science, Qufu Normal University, Qufu, P. R. China;; b Hulunbuir Academy of Inland Lakes in Northern Cold & Arid Areas, Hulunbuir, P. R. China

**Keywords:** Mitochondrial genome, *Motacilla tschutschensis*, phylogenetic analysis

## Abstract

*Motacilla tschutschensis* is a species of small passerine bird belonging to the family Motacillidae. Complete mitochondrial genome of Eastern Yellow Wagtail (*M. tschutschensis*) has been sequenced in this study. The genome is 16,829 bp in length and consists of 13 protein-coding genes, 2 rRNA genes, 1 control region (D-loop), and 22 tRNA genes. The phylogenetic tree was reconstructed using the Bayesian analysis method, indicating that *M. tschutschensis* was closely related to *Motacillalugens* and *Motacillaalba*.

The Eastern Yellow Wagtail (*Motacilla tschutschensis*) is a small passerine bird of the pipit (Anthus) genus, which is classified as Least Concerned (LC) for the Conservation (IUCN) Red List. This species has an extremely large range of habitat, it breeds in NE Siberia and extreme NW North America, winters mainly in SE Asia (MacKinnon et al. 2000).

In this study, the complete mitochondrial genome of the Eastern Yellow Wagtail (*M. tschutschensis*) was sequenced and reported for the first time using muscle tissue obtained from a wild individual died naturally in Hulun Lake National Nature Reserve in Inner Mongolia, China (48.57°N, 117.65°E), and the sample was stored in the Animal Specimen Museum of Qufu Normal University, Qufu, Shandong, China, with the accession number QFA20180047.

The complete mitochondrial genome sequence of the Eastern Yellow Wagtail was deposited in the GenBank with the accession number MN217252 after accurately annotated. The complete mitochondrial genome sequence is 16,829 bp in length and containing 2 rRNA genes, 22 tRNA genes, 13 protein-coding genes, and 1 D-loop region. The total composition is 33.61% C, 30.17% A, 23.70% T, and 14.52% G, and the percentage of A and T (53.87%) is higher than G and C (46.13%). Among these, the ND6 subunit gene and 8tRNA genes (tRNA^Gln^, tRNA^Pro^, tRNA^Ala^, tRNA^Asn^, tRNA^Cys^, tRNA^Tyr^, tRNA^Ser^, and tRNA^Glu^) were encoded in the L-strand, the remaining in the H-strand. The gene arrangement of the *M. tschutschensis*is similar to other Fringillidae species (Lerner et al. 2011; Sun et al. 2016).

The phylogenetic relationship of *M. tschutschensis* and other Passeriformes 17 species were analyzed with the Bayesian inference (BI) method and maximum likelihood(ML) (Ronquist and Huelsenbeck 2003) based on the 12 protein-coding genes, with *Phasianus colchicums*(KT364526) as an outgroup. In this process, we used GTR + I + G as the best-fitting nucleotide substitution model to construct phylogenetic trees with MrModeltest 3.7 (Nylander 2004), and the model and the parameters were then used in the ML and the BI analysis by PAUP 4.0b10 (Swofford 2002) and MrBayes 3.1.2 (Ronquist and Huelsenbeck 2003), respectively. ML tree's reliability was evaluated with a bootstrap test with 100 replicates, and four independent Markov chain runs for 1,000,000 generations. Bayesian analysis showed that *M. tschutschensis* was most closely related to *Motacillalugens* and *Motacillaalba* (Figure 1). The evolutionary relationships of these analyzed species are consistent with previously reported results (Sun et al. 2016). The newly characterized mt genome will help to understand the evolution of Pipits.

**Figure 1.**
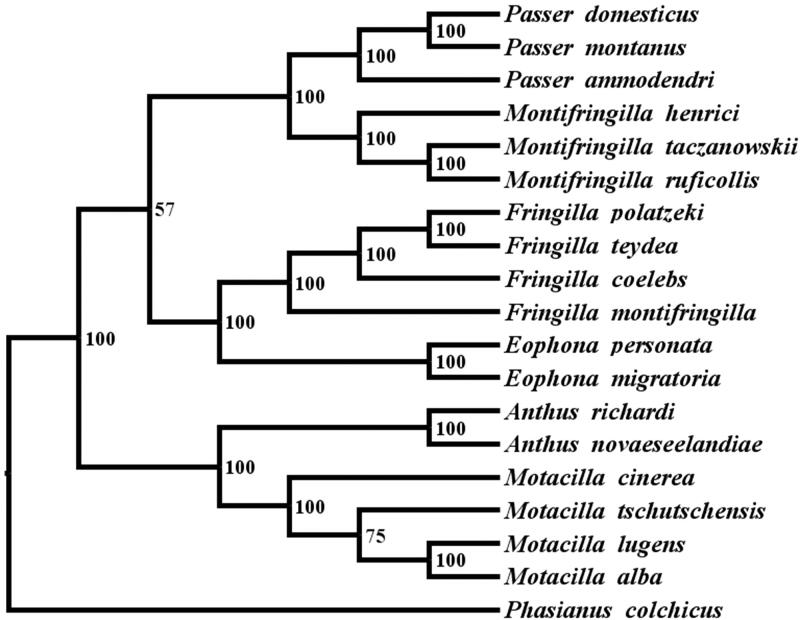
Maximum likelihood (ML) and Bayesian inference (BI) tree based on complete chloroplast genome sequences of 17 species using *Phasianus colchicus* as an outgroup.

All 17 species’s accession numbers are listed as below: *Motacilla lugens* (NC_029703.1), *Motacilla alba* (NC_029229.1), *Motacilla cinerea* (NC_027933.1), *Anthus richardi* (NC_041109.1), *Anthus novaeseelandiae* (NC_029137.1), *Passer domestics* (KM078784.1), *Passer montanus* (KM577704.1), *Passer ammodendri* (NC_029344.1), *Fringilla polatzeki* (NC_031157.1), *Fringilla teydeateydea* (KU705760.1), *Fringilla montifringilla* (JQ922259.1), *Fringilla coelebs* (KM078769.1), *Eophona personata* (KX812499.1), *Eophona migratoria* (NC_031374.1), *Montifringilla henrici* (NC_042414.1), *Montifringilla taczanowskii* (NC_025914.1), *Montifringilla ruficollis* (NC_022815.1).
